# CT-Malaria Detection via Adaptive-Weighted Deep Learning Models

**DOI:** 10.3390/biomedicines14040898

**Published:** 2026-04-15

**Authors:** Karim Gasmi, Moez Krichen, Afrah Alanazi, Sahar Almenwer, Sarah Almaghrabi, Samia Yahyaoui

**Affiliations:** 1 Department of Computer Science, College of Computer and Information Sciences, Jouf University, Sakaka 72388, Saudi Arabia; smalmenwer@ju.edu.sa; 2 Faculty of Computing and Information, Al-Baha University, Al-Baha 65528, Saudi Arabia; k.moez@redcad.org; 3 ReDCAD Laboratory, University of Sfax, Sfax 3038, Tunisia; 4 Department of Information System, College of Computer and Information Sciences, Jouf University, Sakaka 72388, Saudi Arabia; aoalenzy@ju.edu.sa; 5 Department of Information Technology, College of Computing and Information Technology at Khulais, University of Jeddah, Jeddah 21959, Saudi Arabia; ssalmaghrabi@uj.edu.sa; 6 Department of Physics, College of Science, Jouf University, Sakaka 72341, Saudi Arabia; syahyaoui@ju.edu.sa

**Keywords:** SDG 3, malaria detection, optimal algorithm, ensemble learning

## Abstract

**Context:** In numerous low- and middle-income nations, malaria remains a significant issue due to the challenges associated with diagnosing it through thin blood smears. The appearance of images can vary significantly depending on the microscope type, magnification, lighting conditions, slide preparation methods, and staining techniques. Due to the delicate morphology of parasites, false negatives might adversely affect patient care. **Objective:** To achieve optimal outcomes from validation, it is essential to construct a robust and easily replicable process. This pipeline should integrate the optimal elements of classical machine learning and end-to-end deep learning, enhance reliability by pairwise ensembling, and select ensemble weights in a logical, data-driven manner. **Method:** To achieve our objective, we propose two tracks. The initial track encompasses real-time augmentation, convolution-based feature extraction, and the training of calibrated classical classifiers. The second module focuses on training many convolutional networks from inception to completion. Subsequently, we construct paired ensembles and employ a hybrid methodology to select convex weights for combining the findings. This method initially evaluates a set of candidate weights and then refines them to maximise validation accuracy. **Results:** The precision of the two-track architecture consistently improves, transitioning from conventional baselines to end-to-end models. Optimal and consistent enhancements are achieved through weighted ensembling. Utilising optimised fusion reduces the incidence of false negatives for subtle parasites and false positives caused by staining artefacts. This yields an accuracy of 96.35% on the reserved data and reduced variance across folds. **Conclusions:** The integration of augmentation, multiple modelling tracks, and optimal pairwise ensembling yields the highest accuracy in categorising malaria smears. It facilitates further enhancements by incorporating supplementary models, multi-class extensions, and operating-point calibration.

## 1. Introduction

Malaria remains a significant parasite disease worldwide, with elevated morbidity and mortality rates in tropical and subtropical regions [1]. Expert microscopy of Giemsa-stained blood smears is a significant component of clinical protocols. This approach can identify species-level differences, but it is time-consuming, requires considerable expertise, and is susceptible to variations in staining and image quality, complicating large-scale quality-assured screening. Expedited diagnostic methods might accelerate the process; nevertheless, their efficacy can decrease in cases of low parasitemia or infections not attributable to falciparum. Microscopy is a crucial component of numerous programs and laboratory networks [2]. The issue of generalisation for algorithms trained in limited environments is further exacerbated in practice, as images increasingly originate from diverse sources, including standard microscopes with varying optics and configurations, as well as those captured on cell phones [3]. The optical systems of conventional microscopes differ.

In the last ten years, machine learning (ML) and deep learning (DL) have emerged as potentially effective instruments for analysing malaria-related images [4]. These systems operate efficiently in curated datasets; however, they frequently face issues related to stain/device variability and class imbalance [5,6].

Conventional machine learning techniques have demonstrated the feasibility of automated malaria screening; however, they often face challenges in preprocessing and feature generation [7]. Errors in segmentation within packed fields and staining variability can distort color and texture descriptors. This can alter decision boundaries and diminish performance when microscopes or slide preparation techniques are modified [5]. Reviews indicate that the evolution of imaging pipelines requires recalibrating features and thresholds. This not only increases maintenance costs, but also complicates the addition of additional locations [6]. Employing non-imaging machine learning for incidence forecasting underscores that distributions are dynamic and that thorough validation must account for changes in data-generating methods. The data indicate that malaria CAD has become a distribution-shift issue. This implies that models must maintain stability throughout variations in staining, optics, and processes, rather than excelling solely on a singular curated dataset.

Contemporary CNN backbones have significantly reduced discrepancies between automated and expert analyses by directly learning features from pixel data and employing transfer learning to modify pre-trained filters for RBC morphology [3,8]. Comparative and application studies indicate that residual and efficient families offer significant trade-offs between accuracy and efficiency, which is crucial in resource-constrained scenarios [9]. Hybrid multi-branch architectures that integrate EfficientNet-style stems with Dense, Residual, and Inception blocks can more effectively delineate the configuration of local parasites and the context of larger cells. This results in scores deemed superior on publicly available thin-smear datasets. Surveys indicate that the domain shift persists in laboratories, the quality of the labels varies, and insufficient validation can lead to metric inflation if split leakage is poorly managed within the laboratory. Empirical assessments indicate that even reliable backbones may lose robustness beyond the training distribution. This has led to the development of solutions that integrate complementary talents rather than relying on a single model [8,10]. Research on deployment highlights the necessity for compact models and pragmatic augmentation in scenarios with limited memory or computational resources. This illustrates the significance of selecting the appropriate approach for the field [11].

We propose an ensemble that integrates multiple CNN backbones and employs non-uniform weighting to ensure that the most credible experts govern each distribution. For pragmatic purposes, a lightweight metaheuristic may be used to optimise mixture weights on a reserved validation subset. This enables the ensemble to focus on complementary error patterns without altering the backbones themselves. This weighting method works with classification-aware experts that require improved masks and efficient backbones for resource-constrained deployments, thus balancing accuracy, robustness, and efficiency [12]. These design decisions directly tackle the documented issues of domain transitions, acquisition unpredictability, and deployment limitations, while remaining adaptable to more robust frameworks identified in the literature [10].

The remainder of this document is structured as follows: Section 2 offers an extensive review of current studies on malaria detection. In Section 3, we present our proposed framework, which comprises a comprehensive account of preprocessing procedures, data augmentation techniques, feature extraction methods, and an ensemble learning architecture that employs deep classifiers and an optimisation algorithm. Section 4 discusses the experimental setup, encompassing the dataset, evaluation metrics, and baseline configurations. Section 5 presents the empirical findings. This entails evaluating individual models against our ensemble method and assessing the efficacy of the hybrid attributes. In conclusion, Section 5 encapsulates the principal contributions and discusses potential avenues for future research.

## 2. Related Work

Automated malaria diagnosis has advanced through two primary approaches that complement each other effectively. Conventional machine learning (ML) techniques use manually generated features extracted from segmented smear images. These features are small and easily comprehensible; yet they frequently deteriorate as the stain or device changes. Deep learning (DL) algorithms can learn features effectively and typically achieve greater accuracy than alternative methods. However, when used across multiple imaging modalities and laboratories, a single backbone may have limitations. We examine both of these streams. This incremental synthesis underpins our methodology: integrating complementary CNN specialists, optimising their weights, and training them through ensemble learning to preserve and enhance the architecture.

### 2.1. Machine Learning (Classical Pipelines, Features, and Non-Imaging Analytics)

We will commence with the traditional pipeline preprocessing segment, which will be utilised to organise handcrafted features. This section demonstrated not only the feasibility of the idea but also identified the points of brittleness under actual laboratory conditions. Ross et al. (2006) [13] established that integrating automated RBC segmentation with colour and texture descriptors can distinguish between infected and uninfected cells in thin smears. This establishes initial assumptions regarding the efficacy of computer-aided diagnosis (CAD) using curated data.

Tek et al. [14] emphasised parasite-centric attributes computed within segmented cells to overcome the limitations of fundamental thresholding. This resulted in enhanced specificity and demonstrated how little alterations in staining and illumination can influence subsequent attributes. The objective of this was to reiterate the prior instruction. Similarly, Das et al. [15] integrated colour and texture fingerprints with conventional learning algorithms. Their precision was commendable during continuous acquisition; however, it deteriorated with variations in magnification or staining. Subsequent investigations corroborated this inclination.

Muralidharan et al. [16] underscore the significance of the robustness of the feature stage. Subsequently, they assessed various feature selection methods and found that stringent dimensionality reduction significantly improves the stability of SVM/KNN selection with limited data. This indicates that the pre-classifier curation phase may be equally significant as the classifier itself. In conjunction with this optimisation perspective, Gezahegn et al. [5] examined machine learning pipelines and documented the significance of segmentation quality. They found that inadequately segmented or overlapping cells distorted handcrafted descriptors and modified decision boundaries, rendering performance dependent on mask integrity.

In alignment with this perspective, Kunwar et al. [7] presented an extensive classical system that includes illumination stabilisation, RBC segmentation, texture and morphology extraction, and statistical classification. This method demonstrated that textural cues facilitate the differentiation between slides while remaining attuned to variations in slide preparation. This thus rendered robust normalisation and stringent separation methods far more essential. Kalkan and Sahingoz [17], however, began to obscure the distinction between machine learning and deep learning by using learnt filters. They found that as the volume of labelled data increases, learnt representations surpass handcrafted descriptors.

Narayanan et al. [18] conducted a comprehensive comparative evaluation of machine learning and deep learning architectures using cell-level images. The researchers discovered that, when rigorously validated, robust convolutional neural networks (CNNs) outperform conventional models. This breakthrough facilitated the community’s advancement towards representation learning while maintaining conventional preprocessing techniques. Delgado-Ortet and associates [12] demonstrated that an enhanced segmentation front-end utilising deep learning can significantly improve classical detection by concentrating on precise RBC regions. Their research demonstrated the accurate measurement of the impact of mask quality on subsequent accuracy.

Zhao et al. [11] proposed lightweight screening stacks tailored for low-resource settings to address deployment limitations. They demonstrated that pragmatic preprocessing and compact models can provide valuable baselines on constrained hardware, a significant practical consideration despite advancements in deep learning. Paul and Batra [6] conducted a study on concurrent machine learning and deep learning methodologies. They determined that whereas traditional machine learning remains competitive on controlled datasets, it requires significant retuning when methodologies are altered. This highlights a generalisation gap that representation learning seeks to mitigate.

Finally, Odu et al. [19] employed a feature-engineered pipeline, utilising K-means for outlier removal and XGBoost for classification, to model malaria incidence in relation to environmental parameters. This was conducted to demonstrate the prudence required in machine learning with respect to geographical disparities and to emphasise that assessment design must consider the potential for a distribution shift. Their methodology closely resembled that employed in microscopy. Collectively, these machine learning research indicate consistent issues: reliance on segmentation, variability in stains/devices, and the challenge of retuning. Consequently, they advocate transitioning to deep learning to enhance the reliability of data-driven features while adhering to deployment regulations.

### 2.2. Deep Learning (End-to-End RBC Analysis, Hybrids, and Comparative Studies)

In this setting, deep learning reconceptualises computer-assisted malaria diagnosis as end-to-end representation learning. CNN backbones ingest RBC patches and generate hierarchical filters that can detect morphological and texture features induced by parasites. At the outset of this phase, Quinn et al. [20] demonstrated the viability of microscopy-based point-of-care diagnostics. They indicated that learnt features can outperform hand-crafted descriptors, even in constrained circumstances. Liang et al. identified RBC patch classification as a fundamental deep learning task for malaria diagnosis, facilitating the standardisation of experimental protocols with thin-smear images [21]. This was executed similarly.

Dong et al. [22] evaluated various CNN architectures to determine the optimal design efficacy. They concentrated on the functionalities of depth, receptive-field architecture, and the training methodology. Therefore, they implored the community to document systematic ablations alongside key performance indicators. Rajaraman et al. [23] employed pre-trained convolutional neural networks (CNNs), including AlexNet, VGG-16, Xception, ResNet-50, and DenseNet-121, as feature extractors. They demonstrated that transfer learning is effective in scenarios with few labels or when the labels are highly dissimilar. The connection between machine learning and deep learning is very intimate.

Yang et al. [24] extended their research from thin smears to the detection of thick streaks using cellphones. They demonstrated that cellphones can detect thick stains, despite differing optics from those employed in laboratory microscopes. This improvement employs deep learning within realistic field limitations. In a similar vein of problem diversification, Abdurahman et al. [25] created YOLOv3/YOLOv4 to detect parasites in thick blood films. This adaptation denotes the object-detection branch that enhances patch classification. Relatedly, Cınar and Yıldırım [26] conducted a comparative analysis of backbones, revealing a correlation between correctness, architectural depth, and multi-scale design. This resulted in the incorporation of Inception-style and residual-style biases.

Boit and Patil [2] developed EDRI, a hybrid CNN (EfficientNetB2 combined with Dense, Residual, and Inception branches) that achieved an accuracy of approximately 97.7 percent and an area under the curve (AUC) of about 99.76 percent using NIH thin-smear data. This suggests that multi-branch models integrating local and global cues may outperform single-stream backbones. Loddo et al. validated the reliability of residual networks as baselines, necessitating a thorough assessment. In contrast, Alharbi et al. demonstrated that transfer learning and careful tuning may sustain cross-dataset performance, thereby determining which experts should be included in an ensemble [8,10].

Further broadening the technical repertoire, studies explored advanced regularisation and optimisation techniques [27,28], two-stage pipelines that differentiate localisation and classification [29], and Inception-V3 as a multi-scale alternative [30], all supporting the premise that diversity in architectural and training strategies provides advantages amidst variability. Akkasaligar et al. [31] noted that even older architectures such as VGG-16 can remain competitive with appropriate transfer learning during this process. They suggested that ensembles should comprise “modern efficient” and “well-established classic” specialists to achieve a diverse set of errors.

Saha et al. [4] conducted an evaluation of deep learning methodologies from a synthesis perspective, highlighting unresolved challenges. These issues encompass transitions between microscopes or laboratories, an excessive number of classes, inadequate label quality, and constraints on deployment. These challenges require variance reduction through ensembling and privacy-preserving collaboration when data centralisation is impractical. In a study addressing analogous challenges, Jdey et al. [1] contrasted machine learning and deep learning pipelines, emphasising the need for them to be comprehensible, validated, and applicable across multiple sites. They emphasised the significance of ensembles capable of adapting to changes rather than depending on a solitary backbone. Finally, Sarfaraj et al. [9] conducted a comprehensive comparison demonstrating that residual/efficient families consistently exhibit robustness across various datasets. They provided valuable advice on selecting complementary specialists before understanding the optimal weights.

The literature reveals a persistent reality in both areas: individual models struggle during domain transitions, and data governance limits central aggregation. This is the deployment situation under discussion. Consequently, our architecture integrates heterogeneous backbones (residual, efficient, and inception-like), obtains non-uniform mixture weights to ensure that the most reliable experts dominate each distribution, and trains under optimal ensemble learning, as evidenced in prior research.

## 3. Proposed Approach for Malaria Detection

We propose a dual-track pipeline: (A) Data augmentation → CNN feature extraction → classical machine learning classification; and (B) end-to-end deep models → paired ensembles → weighted fusion, with weights optimised by a hybrid Whale Optimisation Algorithm (WOA) and Genetic Algorithm (GA).

**Figure 1:**
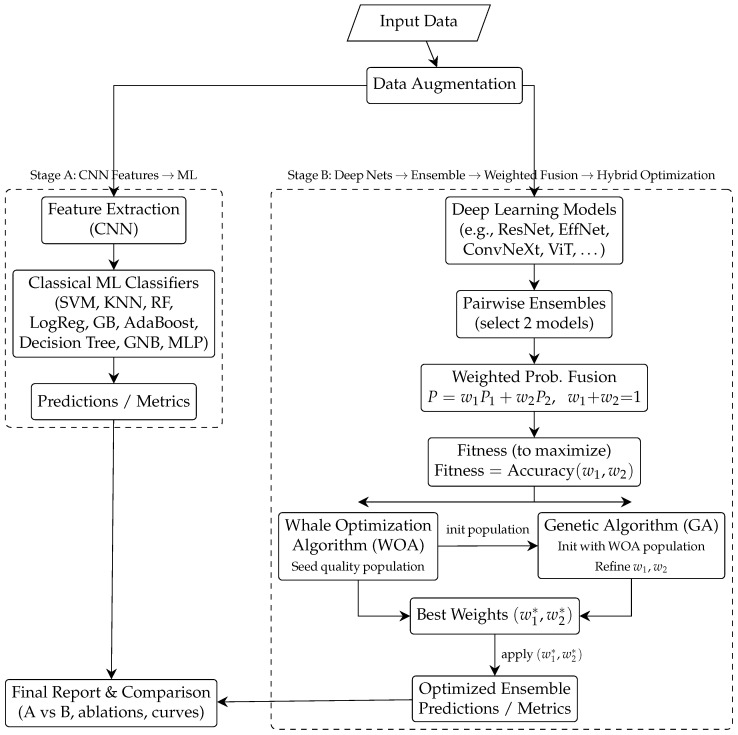
Overview of the proposed hybrid deep learning ensemble framework using WOA for population seeding and GA for refinement.

Our approach, presented in Figure 1, integrates neural representations, traditional machine learning decision functions, contemporary deep architectures, and systematic ensemble optimisation to achieve robust, reproducible malaria detection from thin blood smear images. Significant data augmentation is applied at the beginning of the pipeline to mitigate the effects of class imbalance, microscopy variability, and staining alterations. Track A either immobilises or refines the CNN backbone based on validation signals after extracting discriminative features. It provides a range of machine learning classifiers, including SVM, KNN, Random Forest, Gradient Boosting, Logistic Regression, AdaBoost, Decision Tree, Gaussian Naive Bayes, and MLP, with fixed-length embeddings. In concurrent training, Track B trains multiple advanced deep neural networks from inception to completion, with each network providing calibrated class probabilities. To optimise the models’ disparities, we construct pairwise ensembles and use a convex combination to aggregate the probabilities.

(1) $$\hat{p}=w_{1}{\hat{p}}^{\left(1\right)}+w_{2}{\hat{p}}^{\left(2\right)},\;w_{1},w_{2}\geq 0,\;w_{1}+w_{2}=1.$$

We utilise a hybrid meta-heuristic model to optimise weights, defining fitness as the ratio of validation accuracy to the area under the receiver operating characteristic curve (F1/ROC-AUC). Initially, WOA is employed to cultivate a high-quality populace in proximity to potential places. Subsequently, GA adjusts the weights to converge towards a robust local optimal solution. This incremental optimisation technique accelerates convergence and reduces the likelihood of issues arising from random initialisation. The final report includes ablations (with or without augmentation; single versus ensemble) and comprehensive diagnostics (confusion matrix, ROC/PR curves, Cohen’s kappa, and MCC). The study additionally contrasts Track A and Track B under identical splits.

### 3.1. Dataset Description

We use a public NIH malaria dataset with Infected/Uninfected classes (27,558 images) to ensure comparability and accessibility.

The dataset comprises 27,558 RGB cell images, categorised into two files: “Infected” and “Uninfected.” This dataset is sourced from the National Institutes of Health’s malaria repository (https://ceb.nlm.nih.gov/repositories/malaria-datasets/ accessed on 1 December 2025). It is commonly used as a standard for comparing parasitised cells with non-parasitised cells. To expedite the onboarding process and prevent prolonged downloads from the main website, we utilise a mirrored copy of the website. The images exhibit various alterations in cellular shape, magnification, focus, staining intensity, and artefacts. This diversity compels us to select augmentation and robust modelling. We typically employ the following methodologies: stratified train/validation/test divides, patient-level de-duplication when feasible, and uniform pre-processing. Utilising a public dataset enables objective comparisons with prior research and facilitates the replication of results through standardised protocols and seeds. We maintain comprehensive file lists for each split to ensure that future studies may be consistently replicated with various model families and ensemble configurations.

To ensure a reliable and unbiased assessment of our proposed framework, the dataset is partitioned into three distinct subsets: 70% for training, 10% for validation, and 20% for testing. The training subset is used to optimize the model parameters, while the validation subset supports hyperparameter tuning and early stopping. Importantly, the test subset is kept completely separate from the training process and is not used in any form of model optimization. This strict separation allows the test set to serve as a realistic benchmark, providing an accurate reflection of how the model would perform on previously unseen data in real-world diagnostic scenarios.

### 3.2. Data Augmentation

Augmentation is the process of incorporating additional data types, such as geometry, colour, and texture. This enhances generalisation across slides, microscopes, and staining conditions.

We employ a meticulously designed, balanced augmentation policy customised to our requirements for blood smear imaging. Geometric modifications include refractions, minor scaling or zooming, slight affine perturbations, and arbitrary rotations. Random rotations measure around x degrees. These algorithms replicate genuine acquisition settings, encompassing slide inclination, camera misalignment, and slight focus discrepancies. They precisely replicate these conditions. Photometric modifications, including regulated brightness and contrast adjustments, HSV and saturation adjustments, and per-channel noise, can effectively simulate staining discrepancies and illumination variations while preserving the morphology of chromatin and parasitic structures, which is crucial for precise identification. We also utilise Random Erasing, also known as small occlusions, to reduce the model’s reliance on artificial pixels confined to specific locations. The validation pipeline controls the intensity of these enhancements, as excessive blending can obscure minor parasite characteristics.

### 3.3. Ensemble Machine Learning for Malaria Detection

Classical machine learning ensembles, when utilised with CNN embeddings, establish robust baselines that require less computational effort and are more comprehensible.

We can train several machine learning classifiers with partially related errors by utilising CNN features as static embeddings. This approach is advantageous when computational or memory resources are limited or when elucidating the model’s functionality is crucial, for example, when evaluating feature importance in tree models or analysing margins in support vector machines.

#### 3.3.1. Feature Extraction Based on CNN

We convert images into compact, distinctive vectors with a CNN backbone. Subsequently, we either freeze or slightly adjust the vectors to balance stability and task specificity. Images are resized (e.g., to 224×224), normalised, and then processed by a truncated CNN prior to the classifier head. To obtain a fixed-length embedding (512–2048 dimensions), we use pooled feature maps obtained via global average pooling. To mitigate noise and enhance the efficacy of subsequent machine learning training, we can use a bottleneck (e.g., 256–512 dimensions) with dropout. We standardise embeddings and subsequently store them on disc for reproducible machine learning studies.

#### 3.3.2. Classification Based on ML Models

We train several complementary ML classifiers on the CNN features and calibrate their outputs to obtain reliable probability estimates.

**Support Vector Machine (SVM):** It achieves this by maximising the margin in the embedding space. Utilising RBF kernels facilitates the capture of non-linear boundaries, while class weighting helps maintain balance. Support Vector Machines (SVMs) are robust to small amounts of noise in the samples and often produce distinct decision boundaries.

Primal (soft-margin, linear):

(2) $$\min_{w,b,\xi }\;\frac{1}{2}{∥w∥}^{2}+C\sum_{i=1}^{N}\xi_{i}\;\;s.t.\;\;y_{i}\left(w^{⊤}x_{i}+b\right)\geq 1-\xi_{i},\;\;\xi_{i}\geq 0.$$

Kernel decision:

(3) $$f\left(x\right)=\mathrm{sign}\;\left(\sum_{i}\alpha_{i}y_{i}\;k\left(x_{i},x\right)+b\right).$$

**K-Nearest Neighbours (KNN):**

A non-parametric baseline utilising local neighbourhoods in feature space; this baseline is straightforward and performs effectively when embeddings are appropriately clustered. The distance measurements and k are adjusted to balance bias and variation. In feature space, the distance-weighted vote, sometimes referred to as the majority vote:

(4) $$\hat{y}=\arg\max_{c}\sum_{i\in N_{k}\left(x\right)}\;1\left[y_{i}=c\right],\;w_{i}=\frac{1}{d(x,x_{i})+\varepsilon },\;\;\hat{y}=\arg\max_{c}\sum_{i\in N_{k}\left(x\right)}w_{i}\;1\left[y_{i}=c\right].$$

**Decision Tree:**

Hierarchical splits that are comprehensible and exhibit feature thresholds; although these splits may tend to overfit independently, they are valuable for identifying significant dimensions and for employing weak learners. Split by impurity minimization:

(5) $${\mathrm{Split}}^{=}\arg\min_{s}\sum_{j\in \{L,R\}}\frac{n_{j}}{n}I\left(D_{j}\right)\left(D_{j}\right),\;I_{\mathrm{Gini}}\left(p\right)=\sum_{c}p_{c}\left(1-p_{c}\right),\;I_{\mathrm{Ent}}\left(p\right)=-\sum_{c}p_{c}\log p_{c}.$$

**Random Forest (RF):**

An ensemble of bagged trees that reduces variation through averaging; it accommodates non-linearities and feature interactions, and includes inherent out-of-bag validation for stability assessment. Bagged trees with feature sub-sampling:

(6) $$\hat{P}\left(y=c|x\right)=\frac{1}{T}\sum_{t=1}^{T}{\hat{P}}_{t}\left(y=c|x\right),\;\hat{y}=\arg\max_{c}\hat{P}\left(y=c|x\right).$$

**Gradient Boosting (GB):**

With stringent regularisation (depth, learning rate, and subsampling), sequential trees that address prior residuals perform effectively on tabular embeddings.

Additive model via pseudo-residuals:

(7) $$F_{m}\left(x\right)=F_{m-1}\left(x\right)+\nu \;\gamma_{m}\;h_{m}\left(x\right),$$

with $r_{i}^{\left(m\right)}{=-\partial \ell /\partial F|}_{F_{m-1}}$, and $\gamma_{m}$ line-searched.

**AdaBoost:** Emphasizes previously misclassified samples via adaptive weights; effective when weak learners add orthogonal information.

Exponential loss, reweighting:

(8) $$\alpha_{m}=\frac{1}{2}\ln\frac{1-\varepsilon_{m}}{\varepsilon_{m}},\;\;w_{i}^{\left(m+1\right)}\propto w_{i}^{\left(m\right)}\exp\;\left(-\alpha_{m}y_{i}h_{m}\left(x_{i}\right)\right),\;H\left(x\right)=\mathrm{sign}\;\left(\sum_{m=1}^{M}\alpha_{m}h_{m}\left(x\right)\right).$$

**Gaussian Naive Bayes (GNB):** Fast, probabilistic baseline assuming conditional independence; valuable as a lightweight reference for calibration and error analysis.

Class-conditional independence:

(9) $$P\left(y=c|x\right)\propto P\left(y=c\right)\prod_{j=1}^{d}N\left(x_{j}|\mu_{cj},\sigma_{cj}^{2}\right).$$

**Logistic Regression (LR):** Strong linear classifier with calibrated probabilities; with L1/L2 regularisation provides a sparse, interpretable decision surface.

Linear log-odds with cross-entropy:

(10) $$P\left(y=1|x\right)=\mathrm{sigmoid}\left(w^{⊤}x+b\right),\;L=-\sum_{i}\left(y_{i}\log{\hat{p}}_{i}+\left(1-y_{i}\right)\log\left(1-{\hat{p}}_{i}\right)\right)+\lambda {∥w∥}_{2}^{2}.$$

**MLP Classifier:** A shallow, fully connected network utilising embeddings can capture minor non-linearities without the full complexity of end-to-end CNN training. Feed-forward network with softmax head:

(11) $$h^{\left(l\right)}=\phi \left(W^{\left(l\right)}h^{\left(l-1\right)}+b^{\left(l\right)}\right),\;\hat{p}=\mathrm{softmax}\left(W^{\left(L\right)}h^{\left(L-1\right)}+b^{\left(L\right)}\right).$$

### 3.4. Ensemble Deep Learning for Malaria Detection

We extensively train many contemporary CNN architectures from inception to completion, utilising paired ensembles and optimal weight fusion. We then utilise the distinction among these designs.

We employ a collection of deep models characterised by varying receptive fields, depths, and design paradigms to obtain the most complementary errors. Each model generates a calibrated probability. Various augmentations, seeds, and minor modifications to the training protocol (optimizers/schedules) enhance the complexity. We combine ensemble models, initially using identical weights and subsequently employing optimised weights (Section 3.4.2).

**Generic CNN block.** Convolution → normalization → nonlinearity:

(12) $$Y=\phi \;\left(\mathrm{BN}(X\times K)\right).$$

**ResNet-50/101V2:**

Residual skip connections mitigate disappearing gradients, facilitating the stable training of deeper networks. Bottleneck blocks enhance efficiency; however, multi-stage downsampling balances detail and context, proving advantageous for diminutive parasitic structures. V2 variations enhance pre-activation and normalisation sequencing, resulting in improved gradients [32].

Skip connections ease optimization:

(13) $$y=x+F\left(x;\Theta \right),\;F={\mathrm{Conv}}_{1\times 1}\;\to {\mathrm{Conv}}_{3\times 3}\;\to {\mathrm{Conv}}_{1\times 1}.$$

**VGG-16:**

A basic arrangement of three-by-three convolutions accompanied by maximum pooling. It has several parameters yet provides robust baselines and comprehensible layer activations. It is a straightforward model that performs effectively against contemporary concepts [33].

$$\begin{array}{cc} x_{\mathrm{input}} & \in R^{224\times 224\times 3}\\ \mathrm{VGG}\text{-}16(x) & =\mathrm{Softmax}(W_{3}\;\cdot \;\mathrm{ReLU}\left(W_{2}\;\cdot \;\mathrm{ReLU}\left(W_{1}\;\cdot \;\mathrm{Flatten}\left(C5\right)\right)+b_{2}\right)+b_{3}) \end{array}$$

where the convolutional base (C5) is a sequence of blocks (Conv(F) denotes a 3×3 convolution with F filters, followed by ReLU, and MaxPool denotes 2×2 max pooling):

1. **Block 1:** [Conv(64)]×2→MaxPool
2. **Block 2:** [Conv(128)]×2→MaxPool
3. **Block 3:** [Conv(256)]×3→MaxPool
4. **Block 4:** [Conv(512)]×3→MaxPool
5. **Block 5:** [Conv(512)]×3→MaxPool=C5

And the final classification head uses these steps:

- Flatten(C5): Flattens the final feature map (7×7×512) into a vector.
- FC-1: Fully Connected layer (4096 units, with weights $W_{1},b_{1}$) + ReLU.
- FC-2: Fully Connected layer (4096 units, with weights $W_{2},b_{2}$) + ReLU.
- FC-3: Fully Connected layer (1000 units, with weights $W_{3},b_{3}$) + Softmax activation.

**InceptionV3:** Factorised convolutions and “Inception” blocks with many branches effectively capture features at varying scales. This is beneficial when the parasite’s morphology changes in size and contrast across images [34].

Multi-branch factorization for multi-scale capture:

(14) $$y\;=\;{∥}_{b\in B}F_{b}\left(x\right),\;∥\;\mathrm{is}\;\mathrm{channel}\;\mathrm{concatenation}.$$

**Xception:**

Depthwise separable convolutions separate spatial and channel dimensions, thereby enhancing computational efficiency and improving feature extraction. These convolutions are pretty effective at smearing, producing subtle textural patterns [35,36].

Depthwise separable convolutions:

(15) $$\mathrm{SepConv}\left(x\right)=\mathrm{PWConv}\;\left({\mathrm{DWConv}}_{k\times k}\left(x\right)\right),$$

stacked within residual blocks.

**MobileNetV2:**

Incorporating inverted residuals and linear bottlenecks enables the creation of lightweight models that are sufficiently effective for practical use [37]. Despite its low FLOPs, it maintains considerable accuracy due to depthwise operations.

Inverted residual with linear bottleneck:

(16) $$y=x+{\mathrm{PW}}_{\mathrm{linear}}\;\left(\mathrm{DWConv}(\phi \left({\mathrm{PW}}_{\mathrm{expand}}\left(x\right)\right))\right).$$

**DenseNet121**

Dense connections frequently perform effectively on medical images with limited data, as they enhance feature reuse and gradient propagation. Fine-grained characteristics can transition across levels [38].

Dense connectivity for feature reuse:

(17) $$x_{\ell }=H_{\ell }\;\left([x_{0},\ldots ,x_{\ell -1}]\right).$$

**NASNetMobile:**

The mobile version strikes an effective balance between speed and accuracy, enhancing the ensemble’s architectural diversity. The architecture was identified by neural search [39].

Searched cells (normal/reduction) forming the macro-net:

(18) $$y={\mathrm{Cell}}_{\mathrm{normal}}^{\times n}\circ {\mathrm{Cell}}_{\mathrm{reduction}}^{\times m}\left(x\right).$$

**EfficientNet-B0/B7 & EfficientNetV2-B0/V2-S:** It is remarkable how effectively Fused-MBConv blocks (V2) and compound scaling (depth/width/resolution) harmonise accuracy and efficiency [40]. Elevated resolutions (B7, V2-S) might encompass a more comprehensive background when variations increase [41].

Compound scaling:

(19) $$d=\alpha^{\phi },\;w=\beta^{\phi },\;r=\gamma^{\phi }\;s.t.\;\alpha \beta^{2}\gamma^{2}\approx \kappa ,\;\alpha ,\beta ,\gamma >0,$$

With MBConv/Fused-MBConv and squeeze–excitation.

**ConvNeXt-Large:** A modernized ConvNet with transformer-inspired design cues (large kernels, layer scaling, simpler stems), often yielding excellent performance on vision tasks while remaining CNN-like [42].

Modernized ConvNet with large kernels:

(20) $$y=x+\lambda \;{\mathrm{Conv}}_{7\times 7}\;\left(\mathrm{LN}(\phi (\mathrm{PWConv}(x)))\right).$$

#### 3.4.1. Ensemble Learning

We amalgamate probabilities and create aligned ensembles to mitigate volatility and optimise complimentary strengths.

We initiate the procedure of enumerating paired ensembles and determining the fused probability, denoted as $P=\alpha P_{A}+\left(1-\alpha \right)P_{B}$, utilising deep models independently trained. With a value of α set at 0.5, equal weights establish a robust baseline that enhances calibration and stability due to error decorrelation. We employ a validation split to evaluate each pair and identify combinations that consistently perform optimally, such as EfficientNetV2S and ConvNeXtLarge. Ensembles typically enhance ROC-AUC, PR-AUC, and Specificity without compromising Sensitivity, which is advantageous for screening applications. We additionally affirm that the enhancements remain consistent despite minor test-time augmentations (TTA) and when comparing folds. To prevent overfitting the validation set, we either employ layered validation or maintain a tiny tuning subset exclusively for weight selection. This systematic pairing provides the candidate set for weight optimisation, which will be discussed in the subsequent section.

Given two calibrated predictors with probabilities ${\hat{p}}^{\left(1\right)}$ and ${\hat{p}}^{\left(2\right)}$, we use

(21) $$\hat{p}=w_{1}\;{\hat{p}}^{\left(1\right)}+w_{2}\;{\hat{p}}^{\left(2\right)},\;w_{1},w_{2}\geq 0,\;\;w_{1}+w_{2}=1,\;\hat{y}=\arg\max_{c}{\hat{p}}_{c}.$$

Equal weights ($w_{1}\;=\;w_{2}\;=\;0.5$) serve as a strong baseline; optimized weights are obtained in Section 3.4.2.

#### 3.4.2. Weight Selection for Ensemble Learning Based on Optimization Algorithm

We employ constrained optimisation to select our weights, utilising accuracy as the fitness metric, and apply the F1 and ROC-AUC criterion to resolve ties.

We seek weights $\left(w_{1},w_{2}\right)$ on the simplex defined by $w_{1}+w_{2}=1$, with each $w_{i}$ constrained to the interval [0, 1]. This is accomplished using a model pair (A, B). When multiple weights achieve equivalent accuracy, we select the one with superior accuracy to achieve an optimal balance between precision and recall. The fitness is denoted by Accuracy $\left(w_{1},w_{2}\right)$ for a specific system in the partitioning validation. Our approach emphasises robust operational points rather than overly confident ones. To prevent oscillations, we restrict the step size or mutation range and terminate early when improvements stabilise. The resulting weights are evaluated on an unused test set to obtain an impartial assessment of their efficacy.

(22) $$\max_{w_{1},w_{2}}\;\mathrm{Acc}\left(w_{1},w_{2}\right)\;s.t.\;w_{1},w_{2}\geq 0,\;\;w_{1}+w_{2}=1.$$

#### 3.4.3. WOA and GA Hybridization

The Weight Optimisation Algorithm (WOA) generates a superior weight population, while the Genetic Algorithm (GA) enhances and utilises this population to identify more optimal solutions.

The Whale Optimisation Algorithm (WOA) is inspired by the hunting techniques of humpback whales utilising bubble nets. The objective is to achieve a balance between exploration (seeking numerous alternatives) and exploitation (concentrating on optimal choices). It employs encircling, spiral bubble-net manoeuvres, and random search with diminishing control parameters over time to transition from exploration to exploitation and refine viable solutions. In our context, WOA can rapidly identify intriguing locations on the simplex represented as $\left(w_{1},w_{2}\right)$. This results in the establishment of a diverse and high-quality initial population. A standard, complete WOA runs until full convergence, meaning all $N_{\mathrm{WOA}}$ whales have essentially landed on $w^{★}$. This algorithm is designed to run only for a fixed number of iterations ($T_{\mathrm{WOA}}$). By stopping after $T_{\mathrm{WOA}}$ iterations, the population of whales will have moved into a promising region, but they will not have fully collapsed onto a single point yet.

The Genetic Algorithm (GA) evolves populations over time through selection, crossover, and mutation processes. It accomplishes this by aggregating the weights of highly fit individuals to identify children with superior fitness. The genetic algorithm (GA) excels at local exploitation within favourable basins through elitism and adaptive mutation. It can also escape shallow local optima.

We initiate our hybridisation method by executing WOA for a limited number of iterations to generate a population concentrated on promising weight combinations. This cohort is the inaugural batch utilised by genetic algorithms (GAs) for targeted exploitation. Selection prioritises individuals with superior precision, crossover facilitates the amalgamation of complementary weights, and mutation seeks minor alterations within the simplex while ensuring that $w_{1}+w_{2}=1$. The WOA→GA pipeline minimises the impact of random initialisations on outcomes, accelerates convergence, and establishes stable weights applicable for both validation and testing. The next step involves utilising the selected $\left(w_{1},w_{2}\right)$ to amalgamate probabilities during inference. Subsequently, we present the whole array of metrics, including accuracy, precision, recall/sensitivity, specificity, F1 score, ROC-AUC, PR-AUC, Cohen’s kappa, and the Matthews correlation coefficient (MCC). We also present confusion matrices to aid elucidation.

Unlike existing malaria detection studies that rely on simple majority voting or fixed averaging of CNN models, our method introduces a two-branch architecture that unifies CNN based embeddings with classical ML and diverse deep networks into a single framework. Furthermore, we integrate a hybrid WOA→GA mechanism for continuous convex weight optimization. This model presented by the Algorithm 1. WOA explores wide regions of the weight simplex to identify high quality candidate solutions, while GA refines these solutions through elitism and adaptive mutation. This hybrid strategy improves convergence smoothness, avoids grid search discretization bias, and yields more stable operating points compared with Bayesian or grid based tuning methods.
**Algorithm 1** Hybrid Weight Selection for Pairwise Ensemble (WOA → GA)
**Require:** Validation set V; probabilities ${\hat{p}}^{\left(1\right)},{\hat{p}}^{\left(2\right)}$; sizes $N_{\mathrm{WOA}},N_{\mathrm{GA}}$; iterations $T_{\mathrm{WOA}},T_{\mathrm{GA}}$  1: **Fitness:** for w∈[0,1], $\mathrm{Fit}\left(w\right)=\mathrm{Accuracy}\;\left(\arg\max_{c}{\left[w\;{\hat{p}}^{\left(1\right)}+\left(1-w\right){\hat{p}}^{\left(2\right)}\right]}_{c};\;V\right)$  2: Initialize WOA whales ${\left\{w_{i}^{\left(0\right)}\right\}}_{i=1}^{N_{\mathrm{WOA}}}\subset \left[0,1\right]$ uniformly  3: **for** t=1 to $T_{\mathrm{WOA}}$ **do**▹ WOA exploration/exploitation 4:       Evaluate $\mathrm{Fit}(w_{i}^{\left(t-1\right)})$; let $w^{★}$ be the best  5:       $a\leftarrow 2-2t/T_{\mathrm{WOA}}$; draw $r_{1},r_{2}∼U\left(0,1\right)$; $A=2ar_{1}-a$, $C=2r_{2}$  6:       **for** each whale *i* **do**  7:             **if** |A|<1 **then**▹ encircling best 8:               $w_{i}^{\left(t\right)}\leftarrow w^{★}-A\;\cdot \;\left|C\;\cdot \;w^{★}-w_{i}^{\left(t-1\right)}\right|$  9:             **else**▹ random search 10:                pick random $w_{\mathrm{rand}}$; $w_{i}^{\left(t\right)}\leftarrow w_{\mathrm{rand}}-A\;\cdot \;\left|C\;\cdot \;w_{\mathrm{rand}}-w_{i}^{\left(t-1\right)}\right|$  11:           **end if**  12:           With prob. *p*, spiral update: $w_{i}^{\left(t\right)}\leftarrow \left|w^{★}-w_{i}^{\left(t\right)}\right|\;e^{bl}\cos\left(2\pi l\right)+w^{★}$  13:           Project $w_{i}^{\left(t\right)}\leftarrow \min\left(1,\max\left(0,w_{i}^{\left(t\right)}\right)\right)$  14:      **end for**  15: **end for**  16: Initialize GA population ${\left\{w_{i}\right\}}_{i=1}^{N_{\mathrm{GA}}}$ by sampling from final WOA whales  17: **for** t=1 to $T_{\mathrm{GA}}$ **do**▹ GA exploitation 18:      Evaluate fitness and select parents (e.g., tournament)  19:      Crossover: $w_{\mathrm{child}}\leftarrow \eta \;w_{p1}+\left(1-\eta \right)\;w_{p2}$, η∼U(0,1)  20:      Mutation: $w_{\mathrm{child}}\leftarrow \min\left(1,\max\left(0,\;w_{\mathrm{child}}+\delta \right)\right)$, $\delta ∼N(0,\sigma^{2})$  21:      Elitism: keep top-*k* individuals  22: **end for**  23: **return** $w^{*}\leftarrow \arg\max_{w\in \mathrm{final}\mathrm{GA}\mathrm{population}}\mathrm{Fit}\left(w\right)$  24: $\left(w_{1}^{*},w_{2}^{*}\right)\leftarrow \left(w^{*},1-w^{*}\right)$ 

## 4. Results and Discussion

The proposed framework for malaria detection was rigorously evaluated across four experimental phases, as detailed in the following subsections. Section 4.1 establishes a baseline using prevalent machine learning algorithms, including Support Vector Machines (SVMs), Random Forests (RFs), and k-Nearest Neighbours (k-NN). These classical models could generalise only to a limited set of complex parasitic cell topologies, albeit with a reasonable level of realism. Section 4.2 details how deep learning architectures such as VGG16, ResNet50, and EfficientNet significantly outperformed standard models. These designs might autonomously acquire multiscale spatial and textural features, thereby improving sensitivity and F1-score. Section 4.3 addresses ensemble learning. This entails aggregating the probabilistic outputs of multiple deep models to enhance their stability and robustness. This ensemble technique outperformed others on Receiver Operating Characteristic (ROC) and Cohen’s Kappa, indicating greater model concordance and lower variance. Finally, Section 4.4 presents a method for selecting weights that uses an optimisation strategy to determine each classifier’s contribution to the ensemble. This ensures the optimal equilibrium between precision and generalisation.

To quantitatively assess performance, seven well-known evaluation metrics were employed: Accuracy, Precision, Recall (Sensitivity), Specificity, F1-score, ROC-AUC, and Cohen’s Kappa. Let TP, TN, FP, and FN denote true positives, true negatives, false positives, and false negatives, respectively. The mathematical definitions of these metrics are given as follows:

(23) $$\begin{array}{cc} \mathrm{Accuracy} & =\;\frac{TP+TN}{TP+TN+FP+FN}, \end{array}$$

(24) $$\begin{array}{cc} \mathrm{Precision} & =\;\frac{TP}{TP+FP}, \end{array}$$

(25) $$\begin{array}{cc} \mathrm{Recall}\;\left(\mathrm{Sensitivity}\right) & =\;\frac{TP}{TP+FN}, \end{array}$$

(26) $$\begin{array}{cc} \mathrm{Specificity} & =\;\frac{TN}{TN+FP}, \end{array}$$

(27) $$\begin{array}{cc} F1\text{-}\mathrm{score} & =\;\frac{2\times \mathrm{Precision}\times \mathrm{Recall}}{\mathrm{Precision}+\mathrm{Recall}}. \end{array}$$

The Receiver Operating Characteristic–Area Under the Curve (ROC-AUC) quantifies the model’s ability to distinguish between positive and negative classes by integrating the true positive rate (TPR) and the false positive rate (FPR) over all decision thresholds.

(28) $$\mathrm{AUC}=\int_{0}^{1}\mathrm{TPR}\left(\mathrm{FPR}\right)\;d\left(\mathrm{FPR}\right).$$

Cohen’s Kappa coefficient, which evaluates the agreement between the predicted and actual labels while adjusting for chance agreement, is defined as:

(29) $$\kappa =\frac{p_{o}-p_{e}}{1-p_{e}},$$

where $p_{o}$ is the observed accuracy and $p_{e}$ is the expected accuracy due to random chance.

### 4.1. Machine Learning for Malaria Detection

This phase of the approach employed deep feature extraction alongside standard machine learning algorithms to build a hybrid classification framework. The utilisation of Convolutional Neural Networks (CNNs) as feature extractors enabled the automatic generation of high-level representations from thin blood smear pictures. These representations precisely captured spatial and textural indicators relevant to malaria diagnosis. Subsequently, these distinctive features were integrated into various conventional machine learning classifiers, yielding a CNN-based hybrid architecture. This architecture was designed to leverage the representational capabilities of deep learning while maintaining the interpretability and efficiency of classical models.

The results presented in Table 1 demonstrate the efficacy of the hybrid CNN–ML approach for malaria identification. The CNN-based feature extraction method markedly improved the discriminative power of all traditional classifiers compared to the use of CNN-based feature extraction on manually generated features. Despite the detailed nature of CNN embeddings, simpler techniques such as Gaussian Naïve Bayes and Support Vector Machines (SVMs) generally exhibit lower accuracy, ranging from 63% to 69%. This is due to their inability to represent non-linear decision boundaries fully. Conversely, ensemble methods such as Random Forest and Gradient Boosting achieved accuracies exceeding 82%, non-parasitised and ROC-AUC values of approximately 0.91. These methods were advantageous as they could integrate multiple weak learners and replicate intricate relationships among CNN features. Logistic Regression and the MLP classifier achieved the highest predictive accuracies, with values of 0.8714 and 0.8828, respectively. The Cohen’s Kappa scores were 0.7427 and 0.7656, indicating substantial agreement between the labels. The MLP exhibited the highest overall ROC-AUC (0.9549) and Specificity (0.9343), indicating superior performance in accurately rejecting non-parasitized samples. The results suggest that employing CNN-based deep feature extraction alongside traditional machine learning models yields a robust hybrid solution that harmonises interpretability, efficiency, and predictive performance. Therefore, further exploration of authentic deep architectures and ensemble fusion techniques is essential, as detailed in the following subsections.

We examined many classifiers, including Support Vector Machine (SVM), K-Nearest Neighbours (KNN), Decision Tree (DT), Random Forest (RF), Gradient Boosting (GB), AdaBoost (AB), Gaussian Naïve Bayes (GNB), Logistic Regression (LR), and Multi-Layer Perceptron (MLP). Each model utilised the feature vectors generated by the CNN as input. A stratified 10-fold cross-validation method was used during training to ensure the models’ robustness. The grid search optimisation method was employed to reduce bias and variance by tuning hyperparameters. The objective of this hybrid approach was to evaluate the efficacy of traditional models in utilising CNN-derived embeddings to differentiate between red blood cells infected with parasites and those that are uninfected.

### 4.2. Deep Learning Model for Malaria Classification

We developed and analysed various deep convolutional neural network (CNN) architectures to classify blood smear images as either infected with malaria or not. This phase utilised comprehensive deep learning models that independently execute feature extraction and classification within a unified framework. In the final section, we discus a hybrid CNN–ML pipeline. This differs from independent models. The weights for each model were initially trained on ImageNet and subsequently fine-tuned using the malaria dataset. This was implemented to accelerate convergence and prevent overfitting to a limited set of medical samples. To enhance generality, we employed various data augmentation techniques, including rotation, horizontal flipping, contrast normalisation, and others. The Adam optimiser was employed to improve the procedure. This optimiser had an adjustable learning rate, used categorical cross-entropy loss, and incorporated an early-stopping criterion based on validation performance.

The evaluated models include **ResNet50**, **EfficientNetB0**, **EfficientNetV2B0**, **InceptionV3**, **Xception**, **MobileNetV2**, **DenseNet121**, **NASNetMobile**, **ResNet101V2**, **EfficientNetB7**, **EfficientNetV2S**, **ConvNeXtLarge**, and **VGG16**. These architectures represent various CNN families designed to balance model depth, parameter efficiency, and receptive-field size. Their comparative performance metrics are summarized in Table 2.

**Figure 2:**
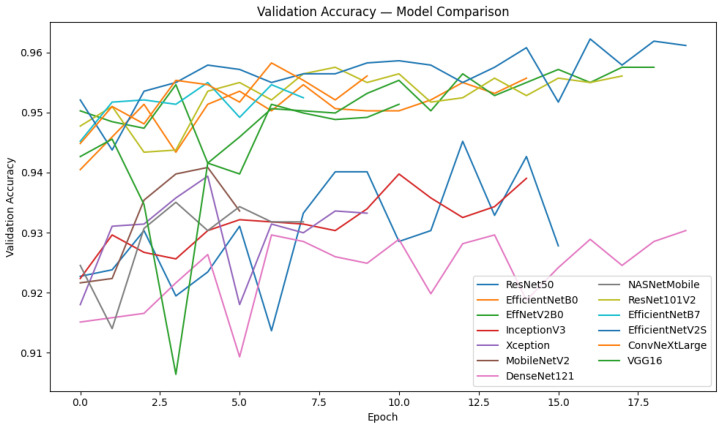
Comparison between deep learning model.

The results in Table 2 and in Figure 2 indicate that all deep CNN architectures performed exceptionally well in classification tasks. All architectures achieved accuracies exceeding 93% and area under the curve (AUC) scores exceeding 0.98. The findings indicate that contemporary CNN designs are proficient at distinguishing individuals with malaria from those without. The ConvNeXtLarge model achieved the highest overall accuracy (0.9579), F1 Score (0.9581), and ROC AUC (0.9918) among all evaluated models. This is demonstrated by its ability to accurately depict intricate cellular classifying malaria images, therebymorphology and hierarchical spatial patterns. The EfficientNet family performed admirably, particularly EfficientNetV2S and EfficientNetB7, which achieved Kappa values over 0.91 and accuracies exceeding 0.954. This demonstrates the efficacy of these models in balancing performance and computational cost.

Legacy architectures such as VGG16, InceptionV3, and Xception exhibit marginally inferior metrics (around 0.93–0.94 accuracy) because of their greater number of parameters and less adaptable scaling techniques. The metrics produced by these architectures were slightly worse. Deep learning effectively reduces both false positives and false negatives, thereby achieving reliable diagnostic sensitivity, as evidenced by the consistently high **Precision** and **Recall** across all models. The inclusion of the PR-AUC, Kappa, and MCC metrics further substantiates the model’s stability and concordance with ground-truth annotations. In conclusion, the findings indicate that deep convolutional neural networks (CNNs) significantly outperform conventional machine learning baselines in the classification of malaria images, hence demonstrating enhanced reliability and generalisability. These findings encourage the implementation of ensemble and optimisation approaches, which will be detailed in the following subsections, to improve robustness and interpretability.

### 4.3. Ensemble Learning for Malaria Detection

Upon concluding our evaluation of the efficacy of several deep learning architectures, we explored ensemble learning to enhance categorisation stability and prediction reliability. The primary objective was to combine the probabilistic outputs (posterior probabilities) of two separately trained CNN models using pairwise averaging. This would enable the acquisition of complimentary feature representations from diverse architectures. This stage is an intermediate fusion level that preserves the diversity of base learners while ensuring computational efficiency.

The final forecast was obtained by averaging the softmax probabilities of both models. We amalgamated each pair of models with equal weights ($w_{1}$ = $w_{2}$ = 0.5) to obtain the final forecast. This straightforward yet effective combination operates on the premise that different designs discern distinct attributes of cell morphology, such as texture, colour, and structural cues. Integrating their assessments helps mitigate overfitting and enhance generality. Table 3 presents a summary of all the pairwise ensembles created during the process. We employed the same test partition and performance metrics for each ensemble. The metrics employed were accuracy, precision, recall, F_1_-score, ROC-AUC, and PR-AUC. This was executed to ensure uniformity across all elements.

The statistics in Table 3 indicate that ensemble fusion resulted in a consistent enhancement in performance relative to standalone deep neural networks. ROC-AUC values beyond 0.99 demonstrated that the majority of pairwise combinations attained accuracies exceeding 95%, hence validating the complimentary characteristics of the aforementioned CNN architectures. The combination of EfficientNetV2S and ConvNeXtLarge achieved commendable performance, with the highest overall accuracy (0.9632) and F1 Score (0.9631). Comparable high-performing pairs, such as EffNetV2B0 + ConvNeXtLarge and ResNet101V2 + EfficientNetV2S, exhibit robust ROC-AUC values (exceeding 0.992) and stable Precision–Recall AUC scores. The findings indicate that these couples exhibit resilience despite the disparity in class status.

It is particularly crucial to emphasise that ensembles comprising models from diverse architectural families, such as residual (ResNet) and compound-scaled (EfficientNet) networks, outperformed those consisting of closely related variants. This indicates the need to present a diverse array of models to reduce the incidence of interconnected errors. Ensemble averaging optimally leverages each network’s capabilities to produce a more stable and generalisable classifier. This is evidenced by improvements across all performance indicators (Accuracy, Precision, Recall, F_1_, ROC-AUC, and PR-AUC). We observed an increase in all performance metrics. The paired findings formed the basis for the subsequent phase of the optimisation procedure, which uses adaptive weight selection to further enhance the ensemble’s performance.

### 4.4. Explainability Analysis Using Grad-CAM

**Figure 3:**
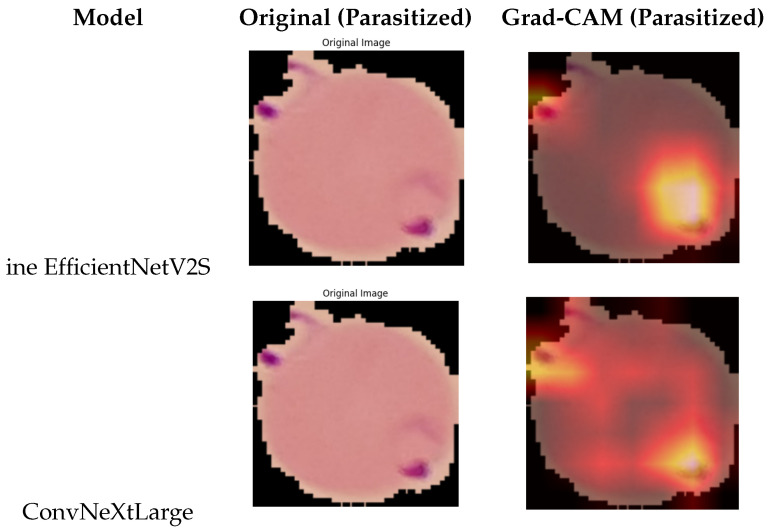
Grad-CAM visualizations for the two best-performing CNN models. The highlighted regions correspond to the morphological locations that most influence the model’s prediction.

To further validate the reliability of the proposed models and ensure that their decisions are guided by clinically meaningful image regions, we employed Gradient-weighted Class Activation Mapping (Grad-CAM) to visualize the spatial attention of each deep network. Grad-CAM produces a heatmap from the final convolutional layer, highlighting the areas that most strongly influence the model’s prediction. As shown in Figure 3, these quantitative findings are further supported by the Grad-CAM visualizations, which confirm that the models focus on biologically meaningful regions.

For Uninfected samples, the Grad-CAM visualizations consistently showed weak and diffuse activation, primarily concentrated around the red blood cell boundary. This indicates that the models do not mistakenly focus on irrelevant intra-cellular regions, and correctly identify the absence of parasite-like structures. The low-intensity response across the interior of healthy cells confirms that no false parasite patterns were detected. In contrast, for Parasitized samples, the heatmaps revealed strong, highly localized activation corresponding to canonical morphological patterns of Plasmodium infection. Bright yellow and red regions typically aligned with chromatin dots, ring forms, and other parasite-induced features within the cell cytoplasm. This demonstrates that the CNNs learned to rely on parasite-specific regions rather than staining artifacts or background noise, which improves the interpretability and trustworthiness of the system. Among all evaluated architectures, **EfficientNetV2S** and **ConvNeXtLarge** exhibited the most concentrated and biologically coherent activation patterns. Their heatmaps showed precise focus on intra-cellular parasite regions, reflecting their superior classification performance. Since these two models constitute the final hybrid ensemble, their Grad-CAM results effectively represent the interpretability of the overall system. As ensemble fusion operates at the probability level, the hybrid model has no convolutional layers of its own; therefore, Grad-CAM cannot be computed directly on the ensemble. Instead, the attention maps of the constituent CNNs serve as the interpretable explanation of the hybrid classifier. Overall, the Grad-CAM visualizations confirm that the proposed models do not act as “black boxes.” Rather, they base their predictions on medically relevant parasite structures, thereby enhancing confidence in the diagnostic decisions produced by the hybrid deep learning framework.

### 4.5. Weight Selection for Ensemble Learning

To surpass simple averaging, we learn the contribution of two top-performing CNNs—EfficientNetV2S and ConvNeXtLarge—via an *optimized Algorithm*. Let $p_{V2S}\left(y\;=\;1|x\right)$ and $p_{\mathrm{ConvNeXt}}\left(y\;=\;1|x\right)$ denote the posterior probabilities for the positive class. The fused probability is a convex combination

(30) $$p_{\mathrm{ens}}\left(y\;=\;1|x\right)\;=\;w\;p_{V2S}\left(y\;=\;1|x\right)\;+\;\left(1-w\right)\;p_{\mathrm{ConvNeXt}}\left(y\;=\;1|x\right),\;\mathrm{with}\;w\in \left[0,1\right].$$

We formulate weight learning as a single-variable constrained optimization:

(31) $$\max_{w\in [0,1]}\;J\left(w\right),\;J\left(w\right)\equiv F_{1}^{\left(\mathrm{val}\right)}\left(w\right),$$

The optimisation approach maintains a set of potential weights, selects those exhibiting optimal fitness, merges parent solutions (crossover) to explore promising regions, and introduces stochastic alterations (mutation) to prevent premature convergence. This search approach is practical in the continuous space w∈[0,1] and yields a solution that approximates the optimal one without employing derivatives.

Table 4 presents a summary of the optimal individuals from each generation of the Genetic Algorithm (GA) retained throughout the adaptive weight optimisation for EfficientNetV2S and ConvNeXtLarge. Each item corresponds to the weight pair $\left(w_{\mathrm{EffNetV}2S},\;w_{\mathrm{ConvNeXt}}\right)$ recognised for its exceptional performance within a population. The results indicate a distinct evolutionary pattern: the initial generations explore a broad spectrum of weight choices, but subsequent generations progressively converge on a small optimal range centred around $w_{\mathrm{EffNetV}2S}\in \left[0.43,\;0.49\right]$. This indicates that the search process is robust and the fitness landscape is substantial.

Throughout all stored populations, accuracy gradually increases, reaching a maximum of 0.9635% at convergence. In the initial generations, it was approximately 0.958 percent. The F_1_-scores for the optimal solution $\left(w_{\mathrm{EffNetV}2S}\;=\;0.449,\;w_{\mathrm{ConvNeXt}}\;=\;0.551\right)$ exhibit a consistent pattern, attaining a value of 0.9635. The ROC-AUC and PR-AUC scores remain exceptionally high, at 0.9918 and 0.9926, respectively. Most of the enhancements result from minor adjustments to the equilibrium among Precision, Recall, and Specificity, rather than from significant changes in separability. The genetic algorithm appears to have identified a globally stable region rather than an overfitted local extremum, as evidenced by the narrow plateau observed at the optimal value.

Furthermore, the evolutionary archive indicates that further generations yield minimal performance variations (<0.001) when the algorithm reaches this plateau, so validating the convergence criterion and demonstrating the reproducibility of the selected weights. The GA-based ensemble tuning is reliable, comprehensible, and reliably convergent. This makes it a reliable method for adaptively weighting multiple deep networks for effective malaria diagnosis. The results of this evolutionary selection indicate that the optimal choice is a harmonious equilibrium between the discriminative capabilities of ConvNeXtLarge and the efficient feature encoding attributes of EfficientNetV2S.

The data in Table 4 indicates that the test performance is evaluated across a substantial quantity of weights. The landscape exhibits a broad optimal zone centred on w≈0.45–0.49. This indicates that ConvNeXtLarge provides a significantly greater contribution. The optimal accuracy occurs at w=0.449 and w=0.469 $\left(\mathrm{Acc}\;=\;0.9635;\;F_{1}\;=\;0.9635\right)$, whereas the highest PR-AUC is seen for w∈[0.408,0.429] (PR-AUC=0.9937). The ROC-AUC will be effectively maximised at approximately 0.9930 across the whole ideal plateau. This indicates that separability will remain robust at all times. The equal-weight baseline w=0.5 demonstrates commendable performance, with an Accuracy of 0.9630 and a F_1_ score of 0.9629. Adjusting it with optimal results in a modest yet consistent increase in both Accuracy and F_1_, while preserving exceptional calibration (PR-AUC).

In our practical application, we utilise the value of $w^{★}=0.449$ (specifically, $w_{V2S}=0.449$ and $w_{\mathrm{ConvNeXt}}=0.551$), which yields Acc=0.9635, $F_{1}=0.9635$, **ROC-AUC** = 0.9930, and **PR-AUC** = 0.9937. The results indicate that optimised weight selection is an effective method for improving the performance of equal averaging and single-model baselines. This results in a robust operating point characterised by balanced Precision and Recall, together with persistent Specificity.

### 4.6. Validation of the Proposed Approach Using a Large Thick and Thin-Smear Dataset

We used the Malaria Dataset from the Nelson Mandela African Institution of Science and Technology (NM-AIST) [43] to do a full evaluation of the adaptive-weighted ensemble architecture we showed. This was to prove that it works and is reliable. This dataset, released in 2024, comprises 3544 images acquired using two smear preparation techniques: thick films (2210 images) and thin films (1334 images). The thick-smear subset enables a thorough, unbiased evaluation because it has a balanced class distribution (Thick-Infected = 1139; Thick-Uninfected = 1071). Even though it is slightly unbalanced (Thin-Infected = 1064 and Thin-Uninfected = 270), the thin-smear subgroup provides additional information on cell shape needed for a full multimodal evaluation.

**Figure 4:**
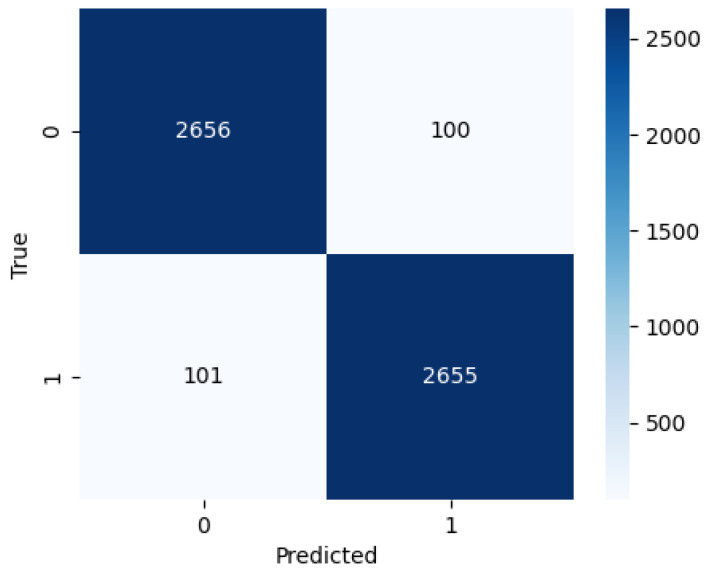
Confusion matrix of the optimized ensemble trained on the combined Thick–Thin dataset.

Table 5 shows examples of thick and thin smear microscopy images that are typical of the total. In thick smears that have been infected, you can usually see little chromatin foci that are darkly stained and clustered parasite remains that are embedded in dense proteinaceous material. You can see parasite parts because these structures are either purple or blue and stand out against a background that is not very clear. Thick smears that are not infected, on the other hand, have diffuse stain deposits, platelet-like particles, or non-parasitic debris without any visible chromatin signals. The NM-AIST dataset contains thin-smear images showing staining patterns different from those seen in typical Giemsa thin films, and was used in the first part of our research. The NM-AIST thin smears don’t always preserve the full shape of each red blood cell, as shown in the example image and in Figure 4.

On the other hand, the smear frequently exhibits partially lysed or deformed red blood cells, resulting in sections that are cloudy, diffuse, and characterised by varying background density. You may see parasite chromatin as small, purple or blue dots scattered across these diffuse regions. However, compared with the best thin-smear preparations, the parasite chromatin within intact erythrocytes is less clear. Uninfected NM-AIST thin smears exhibit heterogeneous staining and amorphous material, rather than clearly defined RBC boundaries. These traits suggest that the “Thin” subset in NM-AIST is more like a weakly lysed preparation than a standard monolayer thin film. Consequently, it serves as an additional component of the thick-smear subset, providing a second smear type that is also visually distinct. The first dataset we used in our research came from thin blood smears; thick films have quite different morphological and optical properties. Thick smears purposefully lyse red cells, creating a granular, heterogeneous matrix characterised by increased stain density and heightened background variability. Thin smears, on the other hand, preserve the shape of red blood cells, making the edges of cells crisper and the shapes of parasites more structured. Images from thin smears show the parasite’s internal phases, whereas images from thick smears show the concentrated parasite chromatin against a thicker background. To ensure the adaptive ensemble model works well across different microscopy modes and preparation techniques, the suggested method should be tested on a specific thick-film dataset. These inherent disparities among smear types emphasise the need to evaluate the proposed procedure.

Thick smears present unique diagnostic challenges due to their disrupted cellular structure, varied background appearance, and increased parasite density. Testing the proposed methodology on this dataset allows us to evaluate its efficacy in both realistic and visually complex scenarios. The data in Table 6 indicates that the adaptive-weighted ensemble frequently outperforms its individual deep components. It has superior accuracy, precision, recall, and agreement coefficients (Kappa and MCC). The results indicate that the ensemble framework effectively integrates complementary deep representations, thereby facilitating the differentiation of thick-film malaria detection.

The trial results utilising the NM-AIST thick-smear dataset illustrate the robustness and efficacy of the adaptive-weighted deep ensemble developed for malaria parasite detection. Thick-film microscopy introduces significant variability in staining density, background texture, and parasite concentration, rendering automated analysis in this domain particularly challenging. Nonetheless, the group consistently performed well across all rating criteria. EfficientNetV2S and ConvNeXtLarge are two complementary backbone architectures, each possessing distinct advantages that synergise effectively. These strengths significantly influenced the observed performance. ConvNeXtLarge offers enhanced specificity and stability across many backgrounds, whereas EfficientNetV2S exhibits increased sensitivity to even the most nuanced parasite characteristics. The enhanced algorithmic weighting may effectively integrate these strengths into a unified decision-making mechanism, resulting in superior overall performance.

The results indicate that the ensemble is not significantly reliant on the feature space of a singular model. Rather, it maximises the interactions among multiple profound representations. In thick-film analysis, where visual intricacy may diminish the accuracy of individual models, this adaptability is crucial as it enhances control over the results. The findings indicate that adaptive ensemble learning may be a reliable method for screening for thick-smear malaria.

### 4.7. Evaluation of the Combined Thick–Thin Training Strategy

To evaluate the efficacy of the proposed adaptive-weighted ensemble in diverse imaging conditions, we performed an extensive experiment using both thick and thin blood-smear pictures from the NM-AIST dataset for concurrent training and assessment. This was executed to demonstrate that the ensemble can accommodate various image conditions. The model undergoes significant alterations in staining, morphology, background structure, and parasite appearance when used in this combinatorial arrangement, compared with research that relies solely on a single smear-preparation technique. Thin smears preserve the integrity of red blood cell membranes and reveal intracellular parasitic species characterised by distinct ring or chromatin structures. Thick smears, conversely, lyse red blood cells, resulting in dense backgrounds and freely floating chromatin puncta. Training the network on this multimodal distribution equips the model with a broader array of discriminative features that can accommodate variations in morphology and staining.

The entire dataset was divided into three segments: 70% for training, 10% for validation, and 20% for testing. This was executed to ensure an adequate mixture of infected and uninfected samples within each subset. Label stratification was maintained throughout the entire process. Data augmentation was utilised just during the training phase to improve generalisation and reduce overfitting. We trained two advanced convolutional backbones, EfficientNetV2S and ConvNeXtLarge, using the same methodology. Subsequently, we integrated their outputs utilising our genetic algorithm (GA)-optimized adaptive weighting method.

Table 7 illustrates the performance of each model and the optimised ensemble. EfficientNetV2S achieved an accuracy of 95.63%, a precision of 98.35%, and a recall of 94.56%. Conversely, ConvNeXtLarge achieved an accuracy of 95.07% and a slightly superior recall. The proposed GA-optimised ensemble regularly outperformed individual models, achieving 96.62% accuracy, 97.25% F1-score, and improved agreement metrics (Kappa = 0.9287, MCC = 0.9291). The results indicate that the ensemble effectively harnesses the strengths of the various models rather than merely averaging their deficiencies.

**Figure 5:**
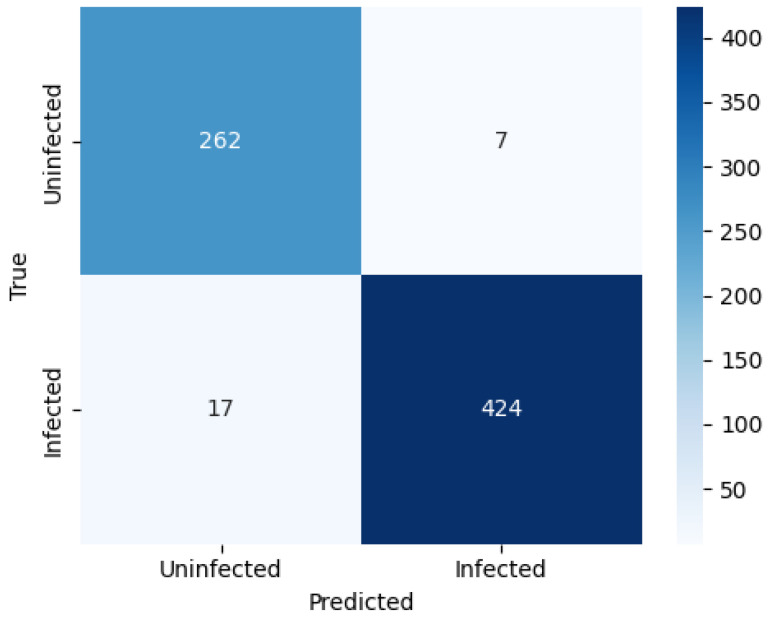
Confusion Matrix for best ensemble learning.

Further insight into the ensemble’s predictive behaviour is provided by the confusion matrix in Figure 5, which shows excellent discrimination between the two classes. Out of 710 test images, the ensemble correctly classified 262 uninfected and 424 infected samples, resulting in only 24 misclassifications (7 false positives and 17 false negatives). The very low false-positive rate is particularly important in clinical screening scenarios, as it minimizes unnecessary follow-up tests on healthy individuals.

These findings strongly indicate that training on a combined Thick–Thin dataset improves the model’s ability to generalize across diverse smear preparation techniques and imaging characteristics. The adaptive-weighted fusion further enhances robustness by integrating complementary recognition patterns from EfficientNetV2S and ConvNeXtLarge. Overall, the results confirm that the proposed ensemble architecture offers a reliable and scalable solution for malaria parasite detection across heterogeneous microscopy data.

### 4.8. Limitations and Future Work

Despite the positive results on the NM-AIST dataset, it is essential to acknowledge certain limitations. Although it is one of the largest publicly accessible datasets of thick-film microscopy, it is limited to data from a single location and employs uniform staining procedures, microscope apparatus, and acquisition parameters. Secondly, thick smears fail to reveal red blood cell morphology, and patterns may vary significantly depending on dye concentration, illumination, and slide preparation quality. The current evaluation may not fully demonstrate performance disparities across laboratories or variations in clinical workflows. Due to the absence of standardised acquisition data, it is unfeasible to examine how these factors influence the model’s behaviour or generate latent biases.

Moreover, while effective, the application of optimisation techniques incurs additional computational costs during the offline weight search phase. Nonetheless, this expenditure occurs only once, and the ultimately deployed ensemble necessitates no additional effort beyond standard inference.

The current study focuses solely on classifying diseases into two distinct categories. In the real world, diagnostic tests may require additional capabilities, such as staging parasites, quantifying them, or differentiating them by species. The existing thick-smear datasets are insufficient for the comprehensive annotations required for these activities.

Future research will investigate multi-centre thick-smear collections, focusing on the evaluation of generalisability across various acquisition contexts: (i) domain adaptation methods to reduce sensitivity to staining and imaging variability; (ii) lightweight model compression for deployment in resource-constrained environments; (iii) extension to species-level detection; and (iv) enhanced interpretability through advanced explainability frameworks beyond Grad-CAM. These represent other domains of inquiry now under investigation.

### 4.9. Comparative Analysis with Existing Malaria Detection Approaches

To contextualise the hybrid and optimised ensemble architecture we developed, we juxtapose our findings with contemporary research on automated detection of malaria using microscopic blood smear images. Utilising handcrafted features such as texture descriptors, colour histograms, or morphological traits in conjunction with classifiers 85% to 90% in conventional machine learning pipelines. Despite their apparent simplicity, these procedures are fraught with numerous issues. For instance, their efficacy depends on the quality of feature engineering, and they cannot capture deep spatial hierarchies in cellular images.

As shown in Table 8, deep learning has significantly enhanced the efficacy of convolutional neural networks (CNNs) such as VGG16, InceptionV3, and ResNet50 in object detection. These CNNs have achieved accuracy rates of 93% to 95%. Although these structures may autonomously extract multiscale visual features, their efficacy may still be compromised by overfitting and differences across datasets. Recent experiments utilising lightweight or compound-scaled designs, such as MobileNetV2 and EfficientNetB0, have demonstrated improved computational efficiency. However, this augmentation typically results in a slight decrease in overall precision and recall.

Compared with these established approaches, our results demonstrate a significant performance improvement. The proposed combination of EfficientNetV2S and ConvNeXtLarge, optimised by a Genetic Algorithm for weight selection, achieves an overall Accuracy of 0.9635, *F*1 score of 0.9635, ROC-AUC of 0.9930, and PR-AUC of 0.9937. This approach surpasses individual CNN models and previous ensembles that used static averaging or voting, which often yield ROC-AUC values below 0.985. The enhancement results from the collaboration of three synergistic components. The components are: (i) CNN-based feature extraction, which identifies robust spatial patterns; (ii) ensemble fusion, which reduces variance and enhances generalisation; and (iii) adaptive genetic weighting, which maintains balanced contributions from the model.

Furthermore, our architecture demonstrates greater consistency across all assessment criteria than hybrid frameworks reported in the literature that combine CNN-extracted features with traditional classifiers. It also demonstrates excellent specificity (exceeding 96%), an essential factor in reducing false positives in medical diagnostics. The results indicate that employing evolutionary optimisation on deep model ensembles represents a significant advancement in automated malaria detection. This advancement yields more reliable diagnoses, improved generalisation across diverse sample types, and a valuable foundation for therapeutic applications in resource-limited settings.

## 5. Conclusions

This paper developed a unified, replicable framework for malaria detection from thin blood smear images, integrating two complementary modelling methods and a systematic ensemble methodology. The initial track utilises fixed convolutional embeddings to train calibrated classical classifiers. This provides robust and comprehensible baselines that utilise a standard augmentation method. The second track trains many end-to-end convolutional networks that are more adept at detecting subtle morphological cues and generalising more effectively than a singular model. We subsequently utilise the discrepancies among models to amalgamate their probabilities using convex weights. We established weight selection as a limited optimisation problem addressed by a hybrid meta-heuristic model. A Whale Optimisation phase identifies suitable candidates, while a Genetic Algorithm phase enhances them. This method consistently enhanced performance and stability across identical divisions of the NIH dataset (27,558 images; Infected/Uninfected), with the optimal ensemble attaining an accuracy of 96.35% on reserved data.

We intend to incorporate federated learning into this pipeline in the future to address data governance, privacy, and representativeness issues at scale. In numerous health systems, digitised smear images are stored separately in hospitals and regional laboratories, and governance regulations complicate their consolidation. A federated protocol would enable training shared models across all participating locations without transmitting raw images. It would employ secure aggregation to maintain the confidentiality of updates and differential privacy to mitigate information leakage. Personalisation layers or meta-learning could enable the global model to function with various staining techniques and microscopes across different locations. Effective aggregation and drift detection would help address the non-IID distributions prevalent in clinical data. To ensure that smaller or resource-constrained centres are not adversely affected during training, it is imperative to implement updates that are readily communicable, establish client selection methodologies, and set objectives that prioritise equity. We want to develop a scalable, privacy-preserving system that learns from a diverse population, complies with legal standards, and enhances real-world generalisation without centralising sensitive patient data. We will integrate the suggested ensembling and weight-optimization techniques with federated orchestration.

## Figures and Tables

**Table 1:** Classical ML models for malaria classification using CNN-extracted features.

Model	Accuracy	Precision	Recall (Sens.)	Specificity	$F_{1}$	ROC AUC	Cohen’s Kappa
SVM	0.6892	0.7313	0.5983	0.7801	0.6582	0.7601	0.3784
KNN	0.7409	0.7465	0.7297	0.7522	0.7380	0.8158	0.4819
Decision Tree	0.7400	0.7417	0.7366	0.7435	0.7391	0.7400	0.4800
Random Forest	0.8260	0.8366	0.8102	0.8418	0.8232	0.9071	0.6520
Gradient Boosting	0.8273	0.8297	0.8237	0.8309	0.8267	0.9068	0.6546
AdaBoost	0.7868	0.7812	0.7968	0.7769	0.7889	0.8678	0.5737
Gaussian Naive Bayes	0.6294	0.6406	0.5893	0.6694	0.6139	0.6744	0.2587
Logistic Regression	0.8714	0.8932	0.8436	0.8991	0.8677	0.9349	0.7427
MLP Classifier	0.8828	0.9268	0.8313	0.9343	0.8764	0.9549	0.7656

Note: We use CNN for feature extraction.

**Table 2:** Malaria classification performance across deep learning models.

Model	Accuracy	Precision	Recall	$F_{1}$	ROC AUC	PR AUC	Kappa	MCC
ResNet50	0.9434	0.9424	0.9445	0.9435	0.9868	0.9867	0.8868	0.8868
EfficientNetB0	0.9516	0.9469	0.9568	0.9518	0.9895	0.9901	0.9031	0.9032
EffNetV2B0	0.9539	0.9523	0.9557	0.9540	0.9905	0.9909	0.9078	0.9078
InceptionV3	0.9343	0.9507	0.9162	0.9331	0.9810	0.9808	0.8687	0.8692
Xception	0.9307	0.9633	0.8955	0.9282	0.9822	0.9814	0.8614	0.8635
MobileNetV2	0.9349	0.9583	0.9093	0.9332	0.9844	0.9849	0.8697	0.8709
DenseNet121	0.9370	0.9207	0.9565	0.9382	0.9824	0.9830	0.8741	0.8748
NASNetMobile	0.9336	0.9561	0.9089	0.9319	0.9806	0.9811	0.8672	0.8683
ResNet101V2	0.9508	0.9748	0.9256	0.9496	0.9894	0.9899	0.9017	0.9028
EfficientNetB7	0.9546	0.9465	0.9637	0.9551	0.9895	0.9904	0.9093	0.9094
EfficientNetV2S	0.9568	0.9605	0.9528	0.9566	0.9910	0.9918	0.9136	0.9137
ConvNeXtLarge	0.9579	0.9530	0.9634	0.9581	0.9918	0.9926	0.9158	0.9159
VGG16	0.9507	0.9484	0.9532	0.9508	0.9866	0.9876	0.9013	0.9013

**Table 3:** Pairwise ensembles ($w_{1}=w_{2}=0.5$) with merged model cells.

First Model	Partner	Acc	Prec	Rec	$F_{1}$	ROC	PR
						AUC	AUC
ResNet50	EfficientNetB0	0.9541	0.9520	0.9565	0.9542	0.9899	0.9903
	EffNetV2B0	0.9537	0.9503	0.9575	0.9539	0.9903	0.9907
	InceptionV3	0.9494	0.9555	0.9427	0.9490	0.9873	0.9875
	Xception	0.9483	0.9643	0.9311	0.9474	0.9880	0.9883
	MobileNetV2	0.9474	0.9580	0.9358	0.9468	0.9882	0.9885
	DenseNet121	0.9450	0.9363	0.9550	0.9456	0.9869	0.9874
	NASNetMobile	0.9487	0.9585	0.9380	0.9481	0.9876	0.9879
	ResNet101V2	0.9539	0.9689	0.9380	0.9532	0.9901	0.9906
	EfficientNetB7	0.9550	0.9491	0.9615	0.9553	0.9903	0.9909
	EfficientNetV2S	0.9570	0.9582	0.9557	0.9569	0.9912	0.9915
	ConvNeXtLarge	0.9585	0.9540	0.9634	0.9587	0.9914	0.9919
	VGG16	0.9501	0.9461	0.9546	0.9503	0.9888	0.9894
EfficientNetB0	EffNetV2B0	0.9563	0.9522	0.9608	0.9565	0.9912	0.9918
	InceptionV3	0.9523	0.9568	0.9474	0.9521	0.9884	0.9886
	Xception	0.9534	0.9647	0.9412	0.9528	0.9891	0.9892
	MobileNetV2	0.9545	0.9606	0.9478	0.9542	0.9894	0.9899
	DenseNet121	0.9507	0.9407	0.9619	0.9512	0.9884	0.9890
	NASNetMobile	0.9537	0.9603	0.9467	0.9534	0.9887	0.9892
	ResNet101V2	0.9570	0.9698	0.9434	0.9564	0.9910	0.9917
	EfficientNetB7	0.9561	0.9509	0.9619	0.9563	0.9911	0.9920
	EfficientNetV2S	0.9581	0.9599	0.9561	0.9580	0.9919	0.9927
	ConvNeXtLarge	0.9588	0.9537	0.9644	0.9590	0.9924	0.9931
	VGG16	0.9546	0.9520	0.9575	0.9548	0.9899	0.9908
EffNetV2B0	InceptionV3	0.9530	0.9595	0.9459	0.9527	0.9891	0.9893
	Xception	0.9514	0.9670	0.9347	0.9506	0.9895	0.9894
	MobileNetV2	0.9545	0.9620	0.9463	0.9541	0.9902	0.9905
	DenseNet121	0.9530	0.9432	0.9641	0.9535	0.9890	0.9897
	NASNetMobile	0.9516	0.9608	0.9416	0.9511	0.9892	0.9894
	ResNet101V2	0.9570	0.9694	0.9438	0.9564	0.9916	0.9921
	EfficientNetB7	0.9585	0.9537	0.9637	0.9587	0.9914	0.9920
	EfficientNetV2S	0.9594	0.9614	0.9572	0.9593	0.9921	0.9926
	ConvNeXtLarge	0.9615	0.9595	0.9637	0.9616	0.9926	0.9932
	VGG16	0.9565	0.9555	0.9575	0.9565	0.9904	0.9913
InceptionV3	Xception	0.9448	0.9647	0.9234	0.9436	0.9852	0.9846
	MobileNetV2	0.9443	0.9609	0.9263	0.9433	0.9870	0.9874
	DenseNet121	0.9454	0.9452	0.9456	0.9454	0.9851	0.9852
	NASNetMobile	0.9436	0.9622	0.9234	0.9424	0.9848	0.9849
	ResNet101V2	0.9508	0.9690	0.9314	0.9499	0.9880	0.9882
	EfficientNetB7	0.9543	0.9573	0.9510	0.9541	0.9886	0.9889
	EfficientNetV2S	0.9548	0.9644	0.9445	0.9544	0.9895	0.9899
	ConvNeXtLarge	0.9577	0.9599	0.9554	0.9576	0.9898	0.9902
	VGG16	0.9481	0.9540	0.9416	0.9478	0.9872	0.9876
Xception	MobileNetV2	0.9396	0.9658	0.9115	0.9378	0.9874	0.9877
	DenseNet121	0.9447	0.9544	0.9340	0.9441	0.9857	0.9859
	NASNetMobile	0.9403	0.9662	0.9126	0.9386	0.9851	0.9852
	ResNet101V2	0.9501	0.9740	0.9249	0.9488	0.9891	0.9893
	EfficientNetB7	0.9517	0.9618	0.9409	0.9512	0.9890	0.9892
	EfficientNetV2S	0.9552	0.9683	0.9412	0.9546	0.9902	0.9904
	ConvNeXtLarge	0.9561	0.9662	0.9452	0.9556	0.9907	0.9910
	VGG16	0.9479	0.9618	0.9329	0.9471	0.9878	0.9884
MobileNetV2	DenseNet121	0.9463	0.9466	0.9459	0.9463	0.9863	0.9870
	NASNetMobile	0.9434	0.9643	0.9209	0.9421	0.9869	0.9871
	ResNet101V2	0.9521	0.9745	0.9285	0.9509	0.9898	0.9903
	EfficientNetB7	0.9556	0.9584	0.9525	0.9554	0.9900	0.9905
	EfficientNetV2S	0.9583	0.9692	0.9467	0.9578	0.9909	0.9913
	ConvNeXtLarge	0.9606	0.9652	0.9557	0.9604	0.9910	0.9915
	VGG16	0.9512	0.9590	0.9427	0.9508	0.9875	0.9883
DenseNet121	NASNetMobile	0.9461	0.9473	0.9448	0.9460	0.9855	0.9861
	ResNet101V2	0.9528	0.9588	0.9463	0.9525	0.9887	0.9894
	EfficientNetB7	0.9523	0.9403	0.9659	0.9529	0.9888	0.9895
	EfficientNetV2S	0.9552	0.9508	0.9601	0.9554	0.9898	0.9907
	ConvNeXtLarge	0.9570	0.9471	0.9681	0.9575	0.9901	0.9910
	VGG16	0.9481	0.9398	0.9575	0.9486	0.9869	0.9878
NASNetMobile	ResNet101V2	0.9534	0.9738	0.9318	0.9523	0.9884	0.9889
	EfficientNetB7	0.9548	0.9587	0.9507	0.9546	0.9891	0.9896
	EfficientNetV2S	0.9585	0.9661	0.9503	0.9581	0.9895	0.9899
	ConvNeXtLarge	0.9597	0.9648	0.9543	0.9595	0.9901	0.9904
	VGG16	0.9505	0.9579	0.9423	0.9501	0.9874	0.9879
ResNet101V2	EfficientNetB7	0.9590	0.9654	0.9521	0.9587	0.9910	0.9918
	EfficientNetV2S	0.9606	0.9718	0.9488	0.9602	0.9918	0.9924
	ConvNeXtLarge	0.9601	0.9686	0.9510	0.9597	0.9923	0.9929
	VGG16	0.9575	0.9705	0.9438	0.9570	0.9900	0.9907
EfficientNetB7	EfficientNetV2S	0.9583	0.9576	0.9590	0.9583	0.9919	0.9928
	ConvNeXtLarge	0.9610	0.9546	0.9681	0.9613	0.9923	0.9932
	VGG16	0.9552	0.9511	0.9597	0.9554	0.9905	0.9913
EfficientNetV2S	ConvNeXtLarge	0.9632	0.9644	0.9619	0.9631	0.9930	0.9936
	VGG16	0.9574	0.9599	0.9546	0.9572	0.9911	0.9919
ConvNeXtLarge	VGG16	0.9617	0.9602	0.9634	0.9618	0.9913	0.9922

**Table 4:** Weighted ensembles of *EfficientNetV2S* and *ConvNeXtLarge* with confusion-matrix counts.

$w_{1}$	$w_{2}$	Accuracy	Precision	Recall (Sens.)	$F_{1}$	ROC AUC	PR AUC	Kappa	MCC
0.449	0.551	0.963534	0.963702	0.963353	0.963527	0.993013	0.993656	0.927068	0.927068
0.469	0.531	0.963534	0.964039	0.962990	0.963514	0.993009	0.993648	0.927068	0.927069
0.429	0.571	0.963353	0.963017	0.963716	0.963366	0.993014	0.993660	0.926705	0.926706
0.388	0.612	0.962990	0.962319	0.963716	0.963017	0.992986	0.993640	0.925980	0.925981
0.510	0.490	0.962990	0.964676	0.961176	0.962923	0.993008	0.993639	0.925980	0.925986

**Table 5:** Representative thick-smear microscopy images illustrating infected and uninfected samples from the NM-AIST dataset.

Class	Image 1	Image 2	Image 3
THICK-Infected	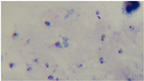	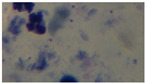	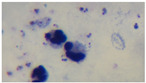
THICK-Uninfected	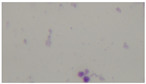	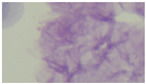	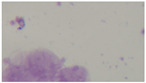
THIN-Infected	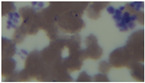	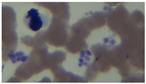	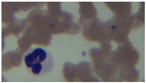
THIN-Uninfected	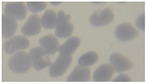	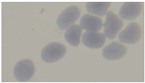	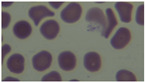

**Table 6:** Performance of the proposed models on the NM-AIST Thick-Smear Dataset.

Model	Acc.	Prec.	Rec.	Spec.	$F_{1}$	ROC-AUC	PR-AUC	Kappa	MCC
EfficientNetV2S	0.9570	0.9906	0.9254	0.9907	0.9569	0.9957	0.9960	0.9141	0.9162
ConvNeXtLarge	0.9434	0.9951	0.8947	0.9953	0.9423	0.9962	0.9966	0.8871	0.8920
**Optimal Ensemble**	**0.9638**	**1.0000**	**0.9298**	**1.0000**	**0.9636**	**0.9985**	**0.9986**	**0.9277**	**0.9301**

Note: The bold number presents the best result.

**Table 7:** Performance of individual models and optimized ensemble on the combined Thick–Thin dataset.

Model	Acc.	Prec.	Rec.	Spec.	$F_{1}$	ROC-AUC	PR-AUC	Kappa	MCC
EfficientNetV2S	0.9563	0.9835	0.9456	0.9740	0.9642	0.9958	0.9975	0.9084	0.9095
ConvNeXtLarge	0.9507	0.9677	0.9524	0.9480	0.9600	0.9911	0.9948	0.8958	0.8960
**Optimal Ensemble**	**0.9662**	**0.9838**	**0.9615**	**0.9740**	**0.9725**	**0.9965**	**0.9979**	**0.9287**	**0.9291**

Note: The bold number presents the best result.

**Table 8:** Comparative Analysis with Existing TB Classification Approaches.

Reference	Accuracy	Recall	Precision	$F_{1}$-Score
Pratiwi et al. [44]	95.83	98	98	96
Prakash et al. [45]	94	94	94	94
Abubakar et al. [46]	94.88	96	94	95
Shekar et al. [47]	96	93	–	–
Shal and Gupta [48]	94.67	–	95.25	–
Dong et al. [22]	95.28	95.50	95.10	–
Rajaraman et al. [23]	95.90	–	–	95.90
**Our Approach**	**96.35**	**96.40**	**96.3**	**96.35**

Note: The bold number presents the best result.

## Data Availability

The data used in this study are openly accessible at the following link: https://www.kaggle.com/datasets/iarunava/cell-images-for-detecting-malaria (accessed on 1 December 2025), https://dataverse.harvard.edu/dataset.xhtml?persistentId=doi:10.7910/DVN/O2WVWA (accessed on 20 March 2026).
